# Junior Doctors' Hours

**Published:** 1992-03

**Authors:** M. D. Read


### From our Correspondents

### JUNIOR DOCTORS' HOURS

The positive approach towards abolishing the Dickensian working conditions of "junior" doctors is most welcome. However, there remains a spectre of the untenable attitude that Consultants are the evil barons of the piece. It is not long since Consultants were referred to as "tyrants" on Radio 4's "Today" programme. The next day the broadsheets were fostering the image of Consultants as dictators followed by a retinue of acolytes who were pressed into the long hours because of unreasonable demands of their "bosses". It may not yet have filtered through to the Department of Health, but times have moved on since Sir Lancelot Spratt strode the corridors of St. Swithens Hospital granting with largesse the occasional half day off per six month appointment. The stereotype of the consultant spending most of his time either doing private practice or playing golf has been well and truly shattered by the recent surveys which show that, despite a maximum contract of 38½ hours, the Consultants **average** working week is 49 hours, **excluding** any on-call committment.

The belief that junior doctors' hours are so long because of Consultants not "pulling their weight" or that junior doctors' hours can be reduced by Consultants picking up their workload shows a remarkable naivety about the workings of the National Health Service. This was underlined by the rather poor response when bids for additional Consultant posts were sought following the Governments's initiative of making an additional 200 Consultant posts available (to cover all specialities in almost 200 districts!).

What no one seems to have picked up is that the leaders of the "juniors" involved in the industrial action of 1975 are now Consultants themselves. Have they undergone a metamorphosis causing them to discard their principles? Of course not — the fact remains that the current problem with junior doctors' hours revolves around two factors, the number of doctors and the hours in a week. The first of these is limited by the Government itself, the second by an even higher authority.

It is true that in a small number of hospitals outdated working practices can be revised to improve the working conditions for the trainess. However, in the majority of cases, particularly in this region, such considerations rarely apply because of the poor staffing levels and "team" working has long been established.

There are a number of measures that can be taken to reduce junior doctors' hours, including shift working and cross cover between specialities. These are not universally applicable, however, especially in the acute specialities. Greater use of locums to cover annual and study leave has been suggested but this is fraught with difficulties as one is left asking who is going to do the locum and how well equipped are they to fit into the department for short terms. There is far more to be said for employing more doctors in the unit to allow internal cover.

There are also a number of measures that can be taken to minimise the adverse effects of the present system, whatever the number of hours worked. There is certainly no need for 3 day weekends which result in the doctor working 84 hours without a break. The guidelines in the recent Department of Health Document state that by 1994 the maximum period worked should be 33 hours during the week or 56 hours at weekend, thus allowing the doctor to work all Saturday, all Sunday, until the end of Monday afternoon. For busy acute specialities this is still an excessive burden and it is possible to arrange rotas so that 33 hours is the maximum worked at any one time. This involves split weekends whereby the doctors would have fewer completely free weekends but it is "safer" for everyone involved. When the trainees are given the option of split weekends or 2 day weekends, there appears to be an even split between those preferring the shorter shifts and those preferring more completely free weekends.

Working a 1 in 4 rota with prospective cover for annual and study leave with a timetable giving a guaranteed half day per week off and a further half day for private study, a registrar would still be working a 72 hour week, for which they will be paid the equivalent of 51 hours pay. Which union would tolerate such working conditions which are already having significant adverse effects on recruitment to the acute specialities? The time has come to face up to the fact that there is no fat left to trim in many districts and what we now need is a major investment in manpower.

M. D. Read

